# Correction: Soil contamination by environmentally persistent free radicals and dioxins following train derailment in East Palestine, OH

**DOI:** 10.1039/d5em90009c

**Published:** 2025-03-03

**Authors:** Myron L. Lard, Sarah E. Eichler, Peng Gao, Kuldeep Singh, Joseph D. Ortiz, Robert L. Cook, Slawomir Lomnicki, Stephania A. Cormier, Jennifer Richmond-Bryant

**Affiliations:** a Department of Chemistry, Louisiana State University Baton Rouge LA 70803 USA; b Department of Biological Sciences, Kent State University Kent OH 44243 USA; c Department of Environmental and Occupational Health, University of Pittsburgh Pittsburgh PA 15261 USA; d Department of Earth Sciences, Kent State University Kent OH 44243 USA; e Department of Environmental Sciences, Louisiana State University Baton Rouge LA 70803 USA; f Department of Comparative Biomedical Sciences, Louisiana State University Baton Rouge LA 70803 USA; g Department of Forestry and Environmental Resources, North Carolina State University Raleigh NC 27695 USA jrbryan3@ncsu.edu

## Abstract

Correction for ‘Soil contamination by environmentally persistent free radicals and dioxins following train derailment in East Palestine, OH’ by Myron L. Lard *et al.*, *Environ. Sci.: Processes Impacts*, 2025, https://doi.org/10.1039/d4em00609g.

The authors regret providing incorrect details of affiliation for Sarah E. Eichler and Slawomir Lomnicki in the original manuscript. The corrected list of author affiliations for this paper is shown herein.

The Royal Society of Chemistry apologises for these errors and any consequent inconvenience to authors and readers.

